# Effect of Poly (Lactic Acid/ε-Caprolactone) Bilayer Membrane on Tooth Extraction Socket Wound Healing in a Rat Model

**DOI:** 10.3390/ma18214956

**Published:** 2025-10-30

**Authors:** Bin Ji, Tingyu Xie, Ikiru Atsuta, Ikue Narimatsu, Yohei Jinno, Akira Takahashi, Mikio Imai, Kiyoshi Koyano, Yasunori Ayukawa

**Affiliations:** 1 Section of Implant and Rehabilitative Dentistry, Division of Oral Rehabilitation, Faculty of Dental Science, Kyushu University, Fukuoka 8128582, Japan; jibin@dent.kyushu-u.ac.jp (B.J.); xietingyu@dent.kyushu-u.ac.jp (T.X.); narimatu.i@dent.kyushu-u.ac.jp (I.N.); jinno315@dent.kyushu-u.ac.jp (Y.J.); mikio@dent.kyushu-u.ac.jp (M.I.); ayukawa@dent.kyushu-u.ac.jp (Y.A.); 2 Division of Advanced Dental Devices and Therapeutics, Faculty of Dental Science, Kyushu University, Fukuoka 8128582, Japan; koyano@dent.kyushu-u.ac.jp; 3 Section of Fixed Prosthodontics, Division of Oral Rehabilitation, Faculty of Dental Science, Kyushu University, Fukuoka 8128582, Japan; a.takaha@dent.kyushu-u.ac.jp

**Keywords:** guided bone regeneration, resorbable membrane, extraction socket, animal model

## Abstract

Guided bone regeneration membranes are essential for bone formation. While non-resorbable membranes require removal surgery, resorbable membranes such as poly (lactic-co-glycolic acid) PLGA are widely used; however, issues with animal-derived components and degradation control have been identified. A novel bilayer membrane composed of synthetic poly (L-lactic acid-co-ε-caprolactone) (PBM) was developed, offering prolonged degradability and elasticity. This study compared the wound-healing effects of PBM and PLGA membranes in vivo and in vitro experiments. In vivo, maxillary molars were extracted from rats, and membranes were placed over the sockets. Healing was evaluated histologically at 1, 2, 3, 4 and 8 weeks. In vitro, oral epithelial cells and fibroblasts were seeded on both sides of PBM. Adhesion and permeability of the membranes were assessed. In vivo, both groups displayed similar mucosal healing. However, PBM preserved a clear bone-soft tissue boundary. In vitro, the surface of PBM supported significantly greater oral epithelial cell adhesion than the reverse side, with no differences for fibroblasts. Both sides of PBM exhibited better protein permeability compared to PLGA. PBM maintained distinct bone-soft tissue separation in rat extraction sockets, suggesting its potential as an effective space maintainer in guided bone regeneration. Further studies are warranted to investigate the mechanisms underlying these favorable properties.

## 1. Introduction

Maintaining sufficient alveolar ridge height and width is critical for preserving natural teeth, enabling successful prosthetic rehabilitation, and ensuring safe implant placement [1]. However, the alveolar bone frequently undergoes significant resorption following periodontal disease progression, prolonged use of ill-fitting dentures, or extended periods after tooth extraction without intervention. This bone loss complicates prosthetic treatment and restricts the options for implant placement [2].

Once resorbed, the alveolar bone does not regenerate spontaneously. To address this, various approaches, including autologous bone grafts and the application of bone substitutes, have been developed to reconstruct alveolar defects [3]. Nevertheless, during the bone regeneration process, the infiltration of soft tissue into the defect site can inhibit osteogenesis. This is largely because fibroblasts (FBs), derived from soft tissues, proliferate more rapidly than osteoblasts [4,5]. Consequently, preventing soft tissue invasion and preserving the regenerative space are critical prerequisites for successful bone augmentation [6,7].

Barrier membranes, a central component of guided tissue regeneration (GTR) and guided bone regeneration (GBR) techniques, have been developed to selectively guide cellular repopulation and promote tissue-specific regeneration. GTR was first introduced in 1982 by Nyman et al. to facilitate periodontal regeneration by creating a space for periodontal ligament-derived cells to migrate, while GBR, introduced in 1988, specifically targets alveolar bone defects by encouraging osteoblast migration and simultaneously preventing soft tissue ingrowth [8,9,10,11].

Membranes used in GTR and GBR are broadly classified into non-resorbable and resorbable types [12,13]. Non-resorbable membranes, such as expanded polytetrafluoroethylene and titanium mesh, provide excellent space maintenance but require a second surgery for removal, which can cause patient discomfort and risks damaging newly formed tissues [14,15]. By contrast, resorbable membranes—primarily made of collagen or synthetic polymers like poly (lactic-co-glycolic acid) (PLGA)—biodegrade over time, eliminating the need for retrieval surgery and improving patient comfort [16,17,18,19].

Despite their advantages, conventional resorbable membranes face challenges, including uncontrolled degradation rates, insufficient mechanical strength, and potential immunogenicity associated with animal-derived materials. Therefore, the development of synthetic, biocompatible membranes with predictable resorption profiles has gained significant attention [20,21,22].

Recently, a novel bilayer resorbable membrane composed of poly (L-lactic ac-id-co-ε-caprolactone) (P(LA/CL); PBM) has been introduced. PBM features a dual-layer structure: a smooth, solid outer layer designed to prevent soft tissue invasion and maintain space, and a porous inner layer that promotes tissue integration and supports osteogenesis. Furthermore, its slow degradation profile and elasticity make it suitable for large defect sites requiring prolonged healing periods [23,24,25].

In this study, we aimed to evaluate the wound-healing effects and biological performance of PBM compared to conventional PLGA membranes, focusing on soft tissue healing, bone regeneration, and cell–membrane interactions through comprehensive in vivo and in vitro analyses.

## 2. Materials and Methods

### 2.1. Materials

Two types of commercially available resorbable membranes were used in this study: a PLGA membrane (GC Membrane, GC Corporation, Tokyo, Japan) and a bilayer membrane composed of P(LA/CL) (PBM; Cytrans Elashield, GC Corporation). Both membranes were utilized in in vitro and in vivo experiments.

### 2.2. Cell Culture

**Figure 1:**
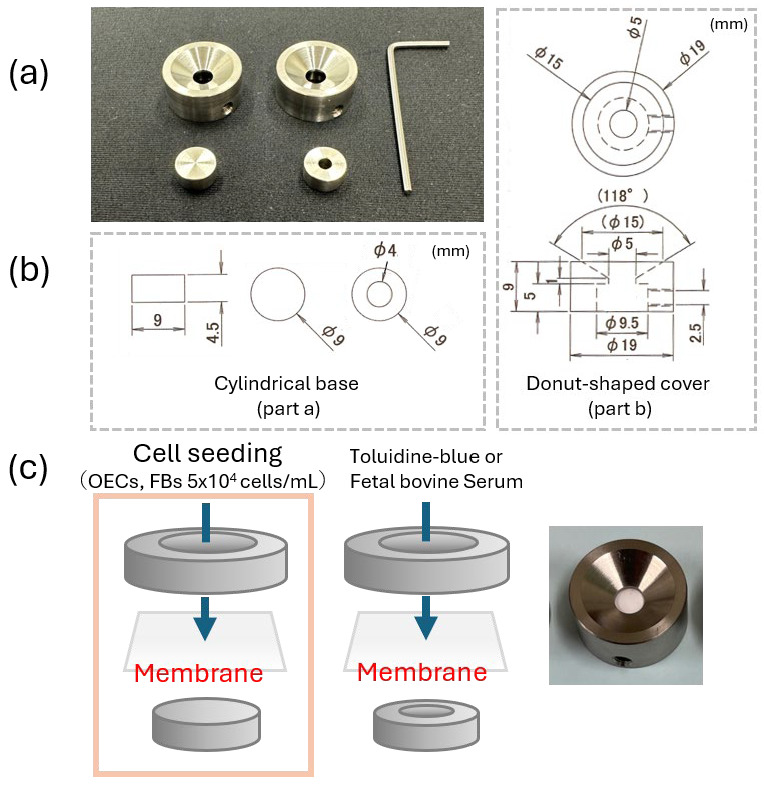
**Custom-designed apparatus for cell culture experiments.** (**a**) Image of the actual device. (**b**) Design schematic. For part a, the leftmost image shows the front view, and the two images on the right show the top views of the two components, respectively. For part b, the upper image shows the top view, and the lower image shows the front view. (**c**) Schematic illustration of membrane placement and an image showing the membrane mounted on the apparatus.

Oral epithelial cells (OECs) and FBs were isolated from rat oral mucosa [26]. Cells were seeded onto either the surface or the reverse side of the PBM membrane at a density of 5 × 10^4^ cells. For seeding, a custom-designed titanium apparatus was used (Figure 1). This device consisted of two parts: a cylindrical base (part a) supporting the membrane and a donut-shaped cover (part b), which is secured to the base by tightening side screws using a small hex wrench. This mechanism firmly locks the membrane in place, preventing displacement and ensuring that cells cannot pass underneath during culture. Cells were cultured under standard conditions (37 °C, 5% CO_2_) for 5 days prior to further analysis.

### 2.3. Scanning Electron Microscopy (SEM)

After 5 days of culture, cell morphology on both sides of the PBM membranes was examined by SEM. Samples were fixed with 2.5% glutaraldehyde, dehydrated through a graded ethanol series, dried, sputter-coated with gold, and observed. In parallel, for quantification of adherent cells, membranes were treated with TrypLE Express (Thermo Fisher Scientific, Waltham, MA, USA) to detach the cells, and the number of cells was counted using an automated cell counter (TC20, Bio-Rad, Tokyo, Japan) [27].

### 2.4. Membrane Permeability Test

**Figure 2:**
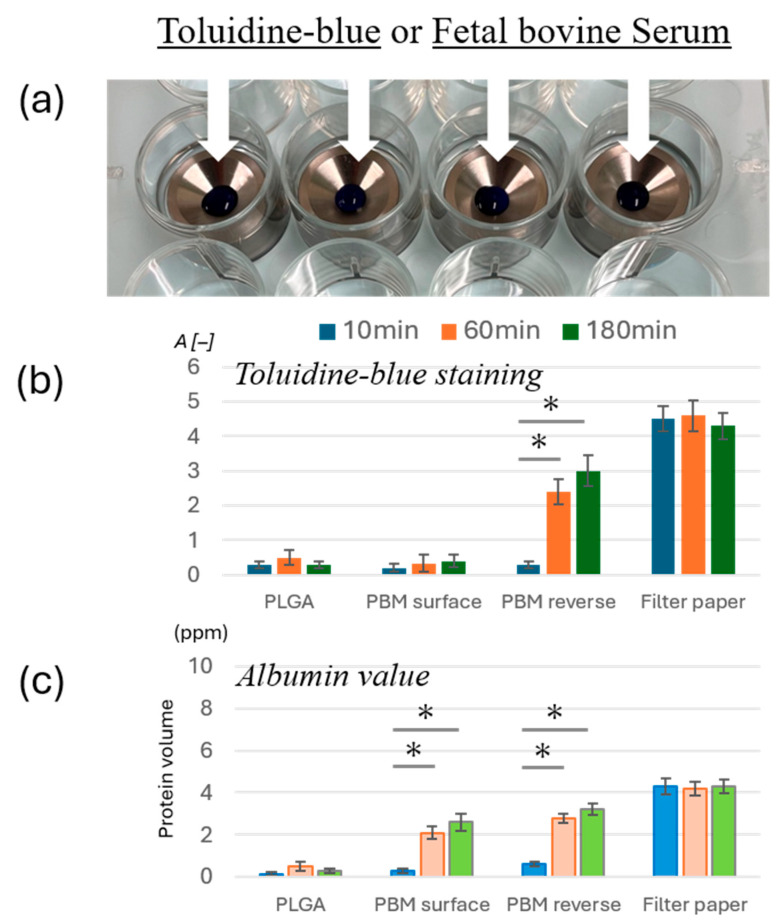
**Permeability comparison between membranes.** (**a**) Image showing the PLGA, PBM surface, PBM reverse, and filter paper mounted on the device. Toluidine-blue and fetal bovine serum were used for permeability testing. (**b**) Toluidine blue penetration through each membrane, evaluated by absorbance measurement (* *p* < 0.05). (**c**) Albumin permeability across membranes, evaluated by protein volume (* *p* < 0.05).

The permeability of membranes was assessed using a modified titanium apparatus designed to hold the membranes in place (Figure 2a). Each membrane (PLGA and PBM surfaces, PBM reverse side, or filter paper as a control) was mounted on the device, which was immersed in an equal volume of distilled water within a 12-well culture plate. Toluidine blue dye or fetal bovine serum (FBS) was applied onto the top of the membranes. After incubation, the degree of penetration was evaluated by determining the absorbance of the underlying water using a spectrophotometer.

### 2.5. Establishment of the Rat Membrane Model

**Figure 3:**
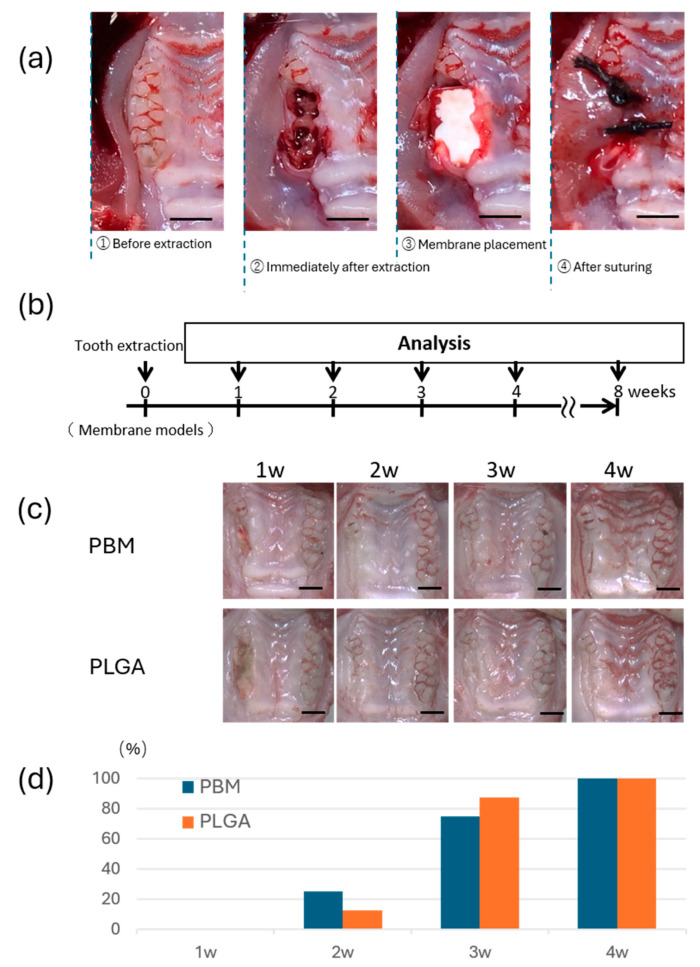
**Mucosal healing in the membrane-covered rat extraction socket model.** (**a**) Step-by-step illustration of membrane placement in the rat oral cavity. Bar = 2 mm. (**b**) Protocol for the in vivo experiment. (**c**) Intraoral images showing the progression of mucosal healing with each membrane. Bar = 2 mm. (**d**) The percentage quantification of mucosal closure was calculated as the proportion of closed samples relative to the total number of samples.

Eighty male Wistar rats (6 weeks, weight 130–160 g, *n* = 8) were used for the in vivo model (Kyudo Co., Tokyo, Japan) [28]. Under general anesthesia, the maxillary right first and second molars were extracted. After extraction, the septum between the extraction sockets was removed using a round bur (CA φ1.0, Dentech, Tokyo, Japan). Each extraction socket was covered with either a PLGA or a PBM membrane. The membranes were sutured to the surrounding mucosa using 4-0 silk thread (two stitches per site) (Figure 3a). Surgery was performed under systemic anesthesia (0.3 mg/kg medetomidine, 4.0 mg/kg midazolam, and 5.0 mg/kg butorphanol), and the animals were maintained in accordance with the guidelines approved by the Animal Care and Use Committee of Kyushu University (Approval Number: A25-240-0). Sutures were removed 3 days postoperatively.

### 2.6. Euthanasia and Sample Preparation

The experimental schedule is shown in Figure 3b; at 1–4 and 8 weeks post-surgery, animals were euthanized by anesthesia overdose. Phosphate-buffered saline perfusion followed by Zamboni’s fixative perfusion was performed. The maxillae were harvested for further analysis. For macroscopic evaluation, specimens were observed using a stereomicroscope. For histological analysis, the specimens were trimmed to preserve the area of interest, fixed in 4% paraformaldehyde for 48 h, and subsequently decalcified in Kalkitox™ solution (Wako, Osaka, Japan) at 4 °C for 24 h. After decalcification, samples were embedded in Optimal Cutting Temperature compound (Sakura Finetek, Tokyo, Japan) and cryosectioned at a thickness of 10 μm using a cryostat.

### 2.7. Micro-Computed Tomography (Micro-CT) Analysis

For radiographic assessment, micro-CT scanning was performed (SkyScan 1076, Bruker, Kontich, Belgium) at 49 kV tube voltage and 201 μA current, with a pixel size of 9 μm. The extraction socket was observed by a coronal plane using micro-CT, with the central part of the second molar on the opposite side serving as a measurement landmark. Bone thickness was evaluated using micro-CT image analysis software (CTAn, Bruker, Kontich, Belgium) [29], and the thickness of the bone was measured from the bottom of the alveolar bone.

### 2.8. Histological Analysis

Frozen sections were stained with hematoxylin and eosin (HE) and Ladewig’s fibrin staining and observed by light microscopy to evaluate soft tissue healing and new bone formation beneath the membranes.

### 2.9. Statistical Analysis

Quantitative data were expressed as mean ± standard deviation. Statistical comparisons between groups were performed using one-way analysis of variance followed by Tukey’s post hoc test for multiple comparisons. A *p*-value of <0.05 was considered to be statistically significant.

## 3. Results

### 3.1. Comparison of OEC and FB Adhesion on PBM Membrane

**Figure 4:**
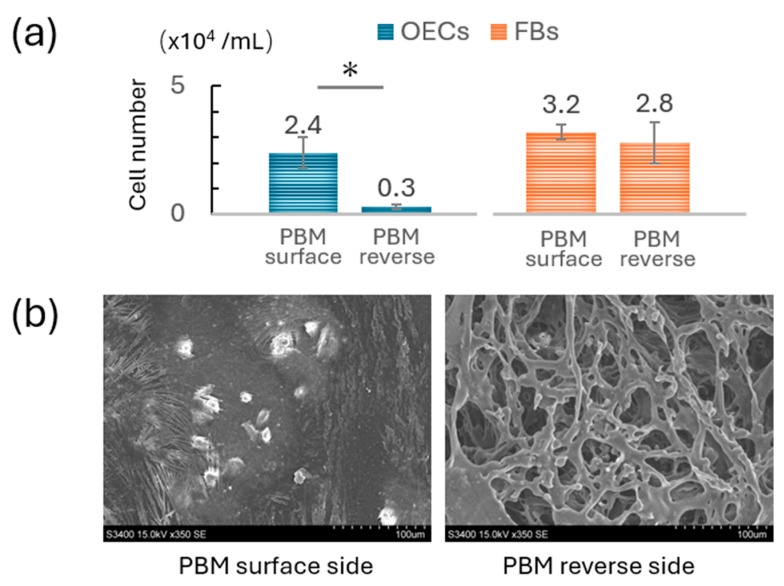
**Comparison of cell adhesion on the surface and reverse sides of the PBM membrane.** (**a**) Quantification of OECs and FBs seeded on each side of the PBM membrane after 5 days of culture (* *p* < 0.05). (**b**) Scanning electron microscopy images of OECs cultured for 5 days on the PBM membrane surface.

Using a custom-designed titanium culture apparatus (Figure 1), OECs and FBs were cultured separately on the surface and reverse sides of the PBM membrane. After 5 days of culture, OECs displayed extensive adhesion on the surface side of the PBM membrane, whereas only minimal adhesion was observed on the reverse side (Figure 4a). Higher magnification SEM images confirmed numerous well-spread epithelial cells with extended lamellipodia on the surface side, whereas few cells were present on the reverse side (Figure 4b). No notable differences in FB adhesion were observed between the two sides of the PBM membrane.

### 3.2. Permeability Differences Between Membranes

Toluidine blue dye and FBS permeability tests were conducted to evaluate membrane barrier properties. Both the PBM surface and PLGA effectively blocked dye penetration, but serum was able to pass through the PBM surface only (Figure 2b,c). By contrast, the reverse side of the PBM exhibited increased permeability over time, similar to that of the filter paper control.

### 3.3. Mucosal Wound Healing over Membranes

Gross examination showed progressive mucosal closure in both the PBM and PLGA groups (Figure 3c). By 3 weeks postoperatively, complete mucosal closure was observed in almost all cases in both groups. Quantitative analysis revealed no statistically significant difference in the rate of mucosal closure between the two groups (Figure 3d).

### 3.4. Bone Formation Beneath the Membranes

**Figure 5:**
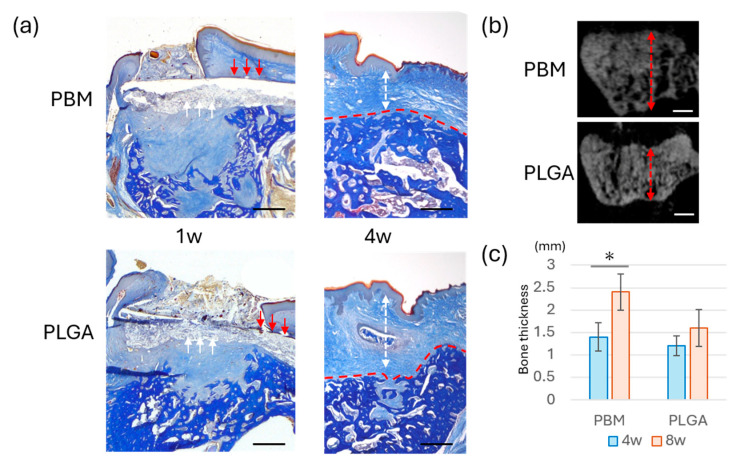
**Histological and radiographic evaluation of bone formation.** (**a**) Ladewig’s fibrin staining of sections at 1- and 4-weeks post membrane insertion. Red arrows indicate the boundary above the membrane; white arrows indicate the boundary below the membrane. Red dashed lines indicate the upper boundary of the bone, and white double arrows indicate the thickness of the connective tissue. Bar = 500 µm (**b**) Micro-CT images of regenerated bone at 4 weeks. Red double arrows indicate bone thickness. Bar = 500 µm (**c**) Quantitative analysis of bone thickness based on the micro-CT data (* *p* < 0.05).

**Figure 6:**
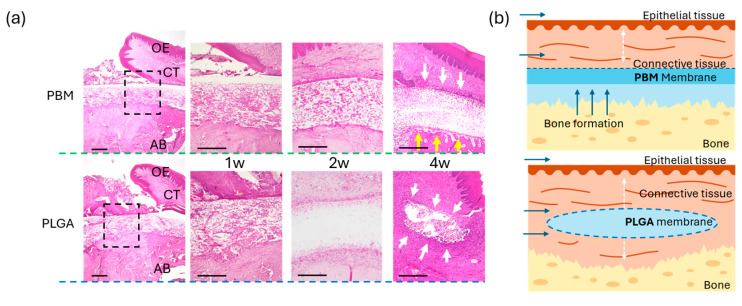
**Healing of tissue surrounding the membranes.** (**a**) Histological sections stained with HE staining, observed at 1-, 2-, and 4-weeks post-surgery. The enlarged image of the black dashed square corresponds to the 1-week specimen. Yellow arrows indicate bone formation; white arrows indicate connective tissue healing. OE: oral epithelial layer, CT: connective tissue, AB: alveolar bone. Bar = 100 µm (**b**) Schematic diagram of tissue healing with the two membranes in the in vivo experiment. White dotted double arrows indicate CT thickness.

Histological analysis at 4 weeks demonstrated differences in the spatial relationship between the membrane and new bone (Figure 5a). In the PBM group, newly formed bone appeared to extend along the membrane. In the PLGA group, a concave defect area was observed beneath the membrane. Micro-CT analysis confirmed these observations (Figure 5b,c); bone thickness was significantly higher in the PBM group than in the PLGA. As shown in Figure 6a, newly formed bone in the PBM group was observed in direct contact with the underside of the membrane. By contrast, the PLGA membrane was surrounded predominantly by connective tissue, with no direct bone contact.

## 4. Discussion

Cell adhesion experiments were performed only on the PBM membrane using a custom titanium apparatus. OECs displayed robust adhesion and spreading with extended lamellipodia on the surface side of PBM, whereas minimal attachment was observed on the reverse side. This indicated that the PBM’s surface side offers a favorable environment for epithelial cell adhesion, likely due to surface topography or hydrophilic properties. Rapid epithelial coverage is critical to prevent postoperative contamination and may support improved early-stage soft tissue healing [30,31]. The lack of adhesion on the reverse side also reflects the directional nature of PBM, emphasizing the importance of correct membrane orientation during surgical placement. Clinically, this directional adhesion underlines the need for clear polarity marking and careful intraoperative handling to ensure the epithelial-facing side is oriented outward.

In the permeability assay using toluidine blue and FBS, both the PBM and PLGA surface sides effectively prevented dye permeation. However, the reverse side of PBM exhibited high permeability comparable to filter paper. This finding underscores a functional limitation of the bilayer PBM design—although the solid surface layer provides strong barrier function, the porous reverse layer may allow undesirable molecular or cellular infiltration if placed incorrectly [32,33]. PLGA, by contrast, is a non-layered uniform membrane, and although permeability testing was limited to one side, it displayed consistent barrier performance. These differences highlight the need for precise membrane orientation when using bilayer membranes like PBM in GBR procedures. Therefore, manufacturers should consider designing refinements or surface treatments to reduce reverse-side permeability while preserving the porous scaffold needed for tissue ingrowth.

Gross examination showed no statistically significant difference in the mucosal healing rate between the PBM and PLGA groups, with both achieving near-complete closure by week three. These results suggested that both materials exhibit adequate biocompatibility to support soft tissue closure. However, considering PBM’s favorable epithelial cell adhesion in vitro, it may offer advantages in more challenging healing environments or in mucosa-compromised patients [34]. Nonetheless, targeted in vivo studies using compromised mucosal models and longer-term follow-up are warranted to determine whether PBM’s in vitro epithelial affinity translates into clinically meaningful improvements in wound stability and reduced complication rates.

Histological and micro-CT evaluation revealed that the PBM group exhibited a larger amount of new bone formation within the extraction sockets compared to the PLGA group. In the PBM group, bone tissue filled the defect space more thoroughly, and a clear boundary was maintained between the membrane and the regenerating bone [35]. This suggested that PBM functions effectively as a space maintainer, promoting osteogenesis while preventing collapse or early soft tissue invasion [36,37,38].

Notably, as shown in Figure 6, new bone was observed in direct contact with the underside of the PBM membrane, suggesting that the membrane surface provided a favorable scaffold for osteogenesis. By contrast, in the PLGA group, the membrane was frequently observed in close contact with connective tissue, and bone regeneration appeared less robust. These findings are in accordance with the principles of GBR, where the exclusion of soft tissue and the maintenance of a secluded compartment for bone precursor cells are considered essential prerequisites for predictable outcomes [9,39]. Previous reports have emphasized that the success of GBR largely depends on three factors: space maintenance, cell selectivity, and wound stability [22,40]. PBM appears to fulfill these requirements more consistently than conventional resorbable membranes such as PLGA, which, although biocompatible, often exhibit faster degradation and weaker mechanical integrity, potentially leading to insufficient space preservation due to their tendency to integrate with soft tissue earlier and thereby cause partial loss of regenerative space during the healing process [24,41,42,43].

Moreover, the clear demarcation observed between PBM and the underlying bone may indicate a biomimetic interaction, where the membrane’s surface topography and chemical properties enhance cellular responses. Studies have shown that collagen-based or structurally anisotropic membranes can promote osteoblast differentiation and angiogenesis while reducing inflammatory cell infiltration [23,24]. In this context, PBM, with its orientation-dependent structure, may provide both osteoconductive and protective functions, thereby creating a more favorable osteogenic environment compared to uniform, isotropic membranes. Taken together, these findings suggested that PBM’s bilayer structure—combining a solid barrier side with a tissue-friendly porous layer—may create an optimal environment for bone healing, provided that its orientation is correct.

To our knowledge, this is the first detailed comparative analysis of PBM and PLGA membranes in an extraction socket model. Our results demonstrate the clinical potential of synthetic bilayer membranes, especially when membrane orientation and structural characteristics are properly considered. Future investigations should further clarify whether this direct bone-to-membrane contact enhances the mechanical stability of the regenerated tissue or facilitates osteoconductive signaling, and whether such interactions can be leveraged to design next-generation membranes with improved regenerative outcomes.

The study results suggested that PBM offers several clinical advantages:

- Enhanced epithelial compatibility for early soft tissue sealing;
- Effective space maintenance for bone regeneration;
- Synthetic and fully resorbable, free from animal-derived components.

However, the performance of PBM is strongly orientation-dependent. Incorrect placement could result in reverse-side exposure to soft tissues, leading to increased permeability and reduced barrier function. By contrast, PLGA membranes, though simpler and less supportive of epithelial attachment, offer a consistent barrier function regardless of placement direction, and may be preferable in cases where membrane orientation is difficult to maintain.

This study has several limitations. First, it used a small animal model with a limited bone defect size and rapid healing capacity, which may not replicate the challenges of human clinical GBR procedures. Second, only short-term healing (up to 4 weeks) was assessed. Long-term membrane degradation, integration, and bone quality outcomes remain unclear.

Future studies should consider the following:

- Evaluate PBM performance in larger animal models with more complex defects;
- Investigate long-term space maintenance and bone maturation;
- Consider design improvements to enhance the barrier function of the reverse side.

## 5. Conclusions

The synthetic bilayer PBM membrane exhibited favorable characteristics for maintaining bone-soft tissue separation and promoting wound healing. These results suggest that PBM may serve as an effective alternative to conventional resorbable membranes for GBR procedures. Further investigations are required to elucidate its long-term clinical applicability.

## Data Availability

The original contributions presented in this study are included in the article. Further inquiries can be directed to the corresponding author.

## Abbreviations

The following abbreviations are used in this manuscript:

PBM	Poly (L-lactic acid-co-ε-caprolactone) Bilayer Membrane
PLGA	Poly (lactic-co-glycolic acid)
GBR	Guided Bone Regeneration
GTR	Guided Tissue Regeneration
OECs	Oral Epithelial Cells
FBs	Fibroblasts
FBS	Fetal Bovine Serum
SEM	Scanning Electron Microscopy
Micro-CT	Micro-Computed Tomography
OCT	Optimal Cutting Temperature (compound)
HE	Hematoxylin and Eosin
OE	Oral Epithelium
CT	Connective Tissue
